# Temporal trends and public health implications of dengue in Guatemala: A decade of challenges and emerging threats (2013–2024)

**DOI:** 10.1016/j.ijregi.2025.100667

**Published:** 2025-05-10

**Authors:** Silvia Patricia Zuniga Veliz, Leticia del Carmen Castillo Signor, Nancy Gonzalez, Joxua Araque, Francisco Hernández, Jorge Ramos, Nevis Nuñez, Francisca Vasquez, Alejandra Barrientos, Edson Jose Adrian Bolanos Lima, Edgar Santos Tejeda, Mayra Lissette Motta Padilla, Anoop Ambikan, Ujjwal Neogi

**Affiliations:** 1 BioBox Guatemala, Guatemala City, Guatemala; 2 Ministry of Public Health and Social Assistance of Guatemala (MSPAS), Guatemala City, Guatemala; 3 Directorate of Epidemiology and Risk Management of the Ministry of Public Health and Social Assistance, Guatemala City, Guatemala; 4 Faculty of Veterinary Medicine and Zootechnics of Guatemala, Guatemala City, Guatemala; 5 Systems Virology Lab, Department of Laboratory Medicine, Karolinska Institute, Stockholm, Sweden

**Keywords:** Dengue, Guatemala, Temporal dynamics

## Abstract

**Background:**

Dengue, a rapidly spreading arboviral disease transmitted by *Aedes* mosquitoes, poses a persistent public health challenge in Guatemala. Rising temperatures increased economic activity, and low population density contribute to the country’s dengue burden, with outbreaks peaking during the rainy season.

**Objectives:**

This study aimed to identify a temporal trend of the dengue epidemic in Guatemala over a decade (2013–2024).

**Methods:**

We collected month- and year-wise dengue case data from 29 provinces between 2013 and October 15, 2024 from the Guatemala’s Ministry of Public Health and Social Assistance database. Additional age-stratified data for individuals under 18 years of age were extracted from the national dengue surveillance databases maintained by the Ministry of Public Health of Guatemala for 2019, 2023 and 2024.

**Results:**

The dengue cases increased significantly from 9357 in 2013 to 117,942 by October 15, 2024, with a notable rise in severe cases and associated mortality. Spatial dynamics also shifted, with the epicenter moving from Huehuetenango in 2019 to central Guatemala in 2024. Children aged 5–9 years were disproportionately affected, with hospitalization rates and case fatalities increasing significantly.

**Conclusions:**

These findings underscore a critical public health crisis, necessitating enhanced surveillance and year-round vector control to mitigate the growing dengue threat in Guatemala.

## Highlights

• Dengue cases rose from 9357 (2013) to 117,942 by October 2024; the severity doubled.
• Dengue epicenter shifted from Huehuetenango (2019) to central Guatemala (2024).
• Children aged under 18 years, especially ages 5-9 years, saw more hospitalizations and fatalities.
• Year-round transmission and rising severity call for better surveillance and control.

## Introduction

Dengue infection, a fast-spreading arboviral disease transmitted by *Aedes mosquitoes* in tropical and subtropical regions, has risen exponentially in recent decades [1]. The recent report from Brazil about the potential transfusion-transmitted dengue virus (DENV) indicated a hidden threat of the dengue epidemic [2]. Dengue fever poses a persistent threat in Guatemala, with transmission peaking during the rainy season. Dengue in Guatemala is a complex public health challenge associated with higher temperatures, increased economic activity, and lower population density [3]. Climate change, particularly, rising temperatures, is projected to exacerbate dengue risks in regions such as El Estor, Iztapa, and Panzós [3]. Climatic conditions such as rainfall play a significant role in the periodicity and severity of dengue outbreaks, especially in southern Guatemala. The first sudden rise in dengue cases in Guatemala was in 2019, with 42,028 cases. In 2023, more than 73,000 cases were reported nationwide, marking a notable rise compared with previous years, and the first National Health Emergency was declared by Guatemala’s Ministry of Public Health and Social Assistance (MSPAS) [4]. However, in 2024, in response to an unusually early and sharp increase in dengue cases, MSPAS declared itself in July [4]. There is no systemic report of temporal dengue upsurge in Guatemala. The aim of the present study was to identify the trend of the dengue epidemic in Guatemala over the past decade.

## Methods

We collected month- and year-wise dengue case data from 29 provinces between 2013 and October 15, 2024 from the MSPAS database [5]. The data set includes cases classified according to the World Health Organization (WHO) 2009 classification system, which is the global standard for dengue diagnosis and case management [6]. This system defines three clinical categories: (i) dengue without warning signs: characterized by fever and at least two of the following: nausea/vomiting, rash, aches and pains, leukopenia, or a positive tourniquet test. (ii) Dengue with warning signs includes all features of dengue without warning signs plus any of the following warning signs: abdominal pain or tenderness, persistent vomiting, fluid accumulation, mucosal bleeding, lethargy or restlessness, liver enlargement (>2 cm), or laboratory evidence of increased hematocrit with a rapid drop in platelet count. (iii) Severe dengue is defined by at least one of the following: severe plasma leakage leading to shock or fluid accumulation with respiratory distress, severe bleeding, or severe organ impairment (e.g. aspartate transaminase/alanine transaminase ≥1000, central nervous system involvement, myocarditis). In response to the increased impact of dengue among children in 2024, additional age-stratified data for individuals under 18 years of age were extracted from the national dengue surveillance databases maintained by the Ministry of Public Health of Guatemala. These data sets, covering the years 2019, 2023, and 2024, were reviewed separately to identify temporal trends, case distribution by age group, and severity patterns in the pediatric population. The data are available in figshare (https://doi.org/10.6084/m9.figshare.28933982).

The bar graphs and line plots were created using ggplot2 v3.5.1, and the map was created using R packages rnaturalearth v1.01 and rnaturalearthdata v1.0.0 (https://cran.r-project.org/web/packages/rnaturalearth/index.html). A total of 23 provinces were included on the map. Province Guatemala Central, Guatemala Nor-Occidente, Guatemala Nor-Oriente, and Guatemala Sur were combined into Guatemala, whereas Petén Norte, Petén Sur Occidental, and Petén Sur Oriental were considered as single province due to the lack of availability of map data in the R package.

## Result

**Figure 1:**
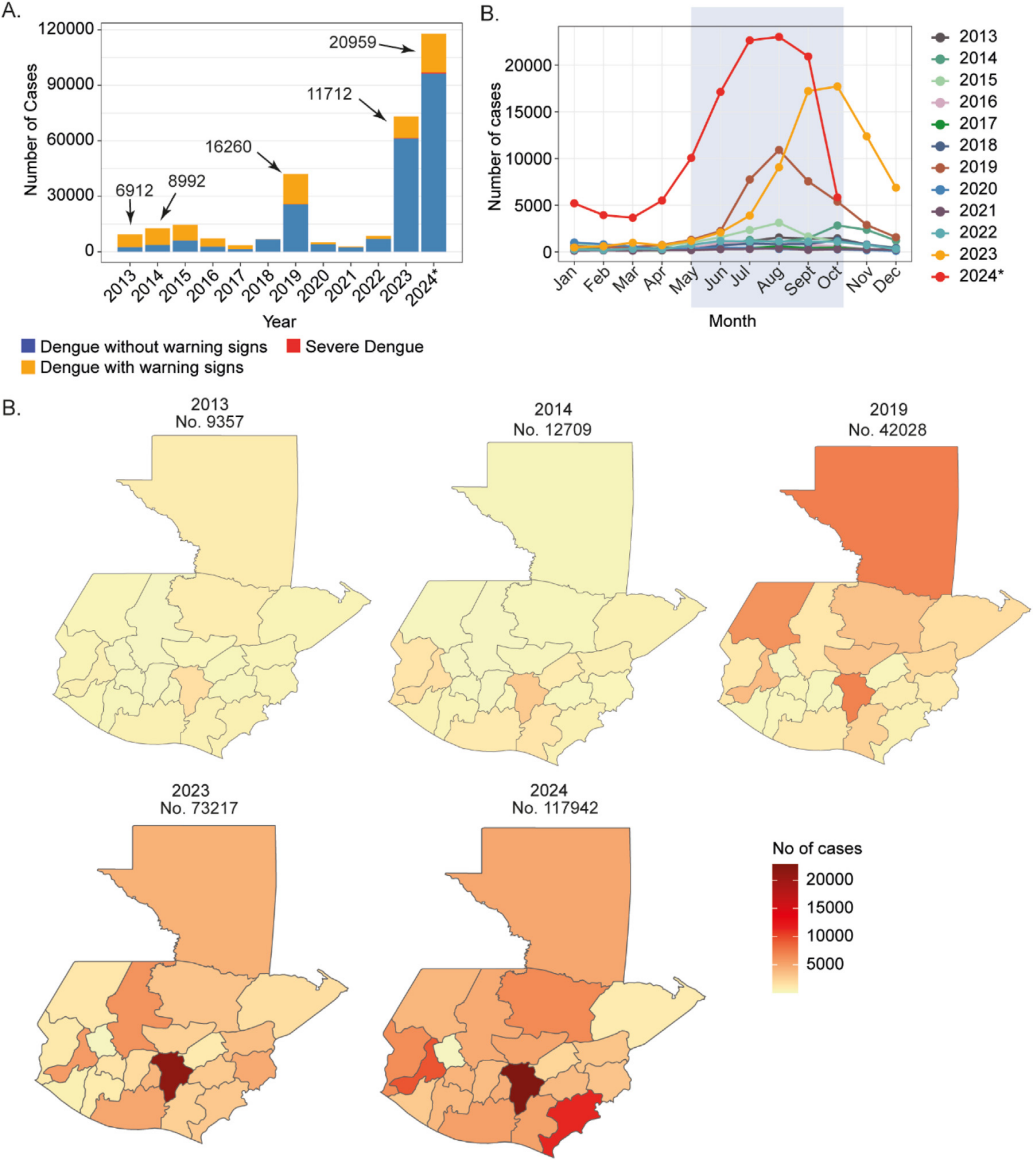
(a) Stacked bar graph showing the yearly occurrence of dengue cases grouped by disease categories. (b) Line plot showing the monthly occurrence of dengue cases for each year from 2013. (c) The number of dengue cases in each Guatemala province. The total number of dengue cases in each year is labeled above. A total of 23 provinces were included on the map. Provisions Guatemala Central, Guatemala Nor-Occidente, Guatemala Nor-Oriente, and Guatemala Sur were combined into Guatemala, and the same was done for Petén Norte, Petén Sur Occidental, and Petén Sur Oriental due to the lack of availability of map data in the R package.

There was a gradual increase in the number of dengue cases from 9357 in 2013 to 117,942 in 2024 (until October 15). The highest cases of dengue with warning signs were reported in 2019, with 16,260 (of 42,024, 38.6%), whereas the incidence was reduced in 2023 to 11,712 (of 73,217, 16%). However, in 2024 (until October 15), there was 1.6 times increase in cases, to 117,942 and 1.9 times in dengue with a warning sign (20,959 cases) (Figure 1a). There was also an increase in mortality to 658 in 2024 from 360 cases in 2023. The number of reported cases in the month indicates the epidemic has expanded to around the year (Figure 1b). In 2019 and 2023, the increase in the dengue season started in July and peaked in August and October, respectively. However, in 2024, the season started in January, with >9000 cases reported. There has also been a change in the spatial dynamics of dengue cases. In 2019, the highest cases were reported from Huehuetenango (6185 cases) in the western part of the country, whereas in 2024, it was central Guatemala in the city area (20,933 cases), potentially due to a better surveillance system (Figure 1c).

**Figure 2:**
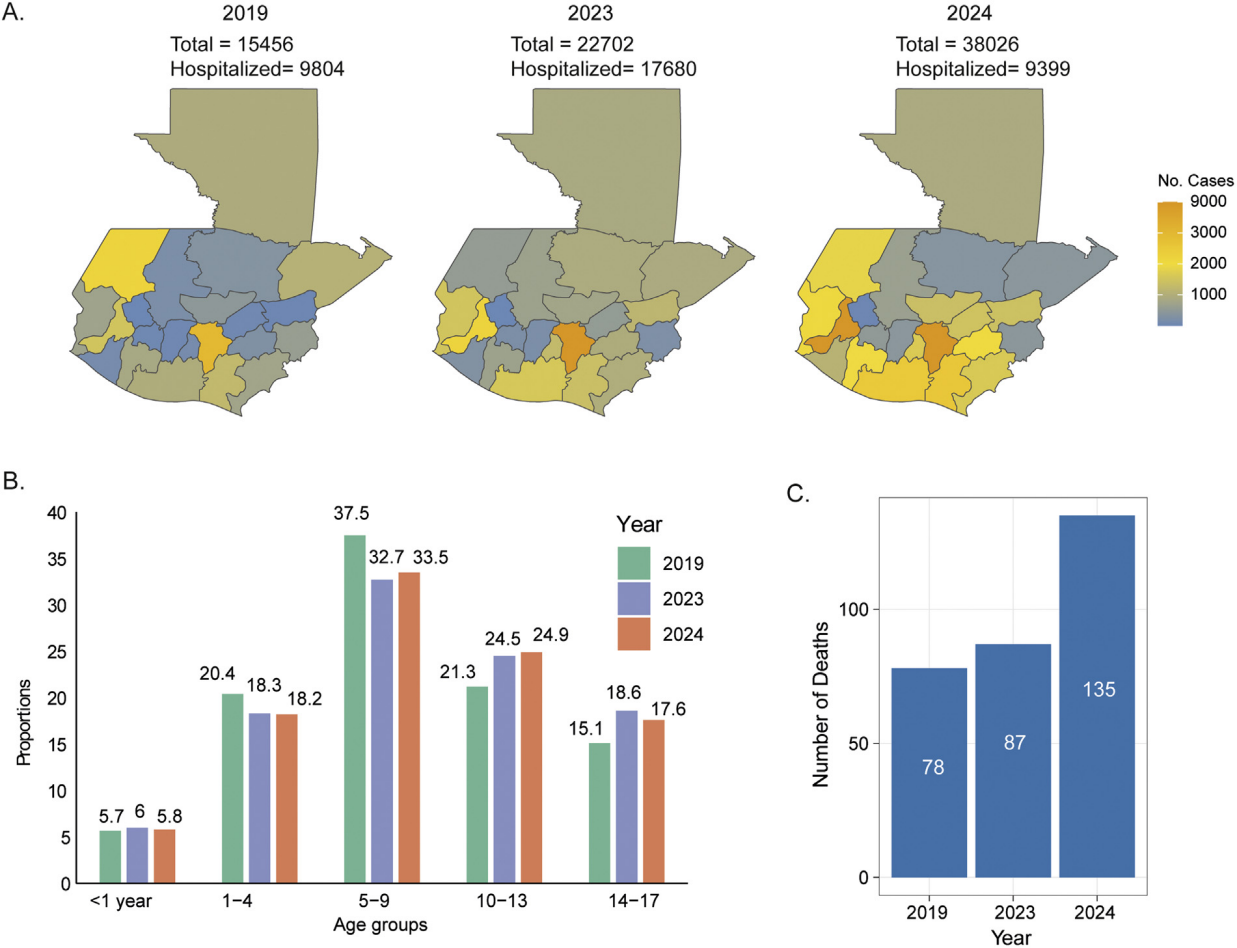
(a) The number of dengue cases in each Guatemala province under 18 years. The total number of dengue cases and the hospitalized cases in each year are labeled above. (b) Year-wise bar plots indicated the proportion of dengue cases in different age groups in 2019, 2023, and 2024. (c) The number of death cases among hospitalized patients.

Among the population, in 2024, one of the major impacts is the children below 18 years of age. In 2019, among the total cases, 42,028, 15,456 (36.7%) were children under 18 years of age, of whom 63.4% (n = 9804) needed hospitalization. A similar pattern was observed in 2023, with 22,702 cases and 17,680 hospitalizations. However, in 2024, the number of hospitalizations was 24.7% (n = 9399) despite having 38,026 cases (Figure 2a). The spatial pattern looked the same as the overall incidence (Figure 2a). Among the different age groups, those aged 5-9 years were more impacted in all 3 years (Figure 2b). The case fatality rate among hospitalized children increased significantly from 0.79% (n = 78) in 2019 to 1.4% (n = 135) in 2024 (Figure 2c), indicating an alarming pattern of severity in the children.

## Discussion

In this study, we reported that the dengue cases in Guatemala increased significantly from 9357 in 2013 to 117,942 by October 15, 2024, with a notable rise in severe cases and associated mortality, which nearly doubled from in 2024 compared with the previous years, demonstrating a critical public health challenge. The epidemic, previously seasonal, now persists year-round, with the 2024 outbreak starting as early as January. Spatial dynamics shifted, with the highest cases moving from Huehuetenango in 2019 to central Guatemala in 2024, likely due to improved surveillance. Pediatric populations, especially children aged 5-9 years, are disproportionately impacted, with rising hospitalizations and fatalities emphasizing the growing severity of dengue among the youth.

Since 2008, in Brazil [7] and 2010 in Colombia [8], cases of dengue, severe dengue, and dengue-related deaths have been occurring more frequently among children aged under 15 years [9]. Moreover, the co-circulation of DENV serotypes 1-4 (DENV-1, DENV-2, DENV-3, and DENV-4) were reported in Guatemala [10], which may significantly increase the risk of severe dengue due to a phenomenon known as antibody-dependent enhancement [11]. In cases of secondary dengue infections with a different serotype, non-neutralizing antibodies from a previous infection can facilitate viral entry into host cells, enhancing viral replication and exacerbating the disease. Notably, the co-circulation of these serotypes, as reported in Guatemala, significantly amplifies the likelihood of secondary infections and the associated risk of severe manifestations of dengue. Moreover, socioeconomic factors, population density, economic activity, and the environmental factor of average minimum yearly temperature significantly predicted dengue risk in Guatemala [3].

In Guatemala, dengue surveillance and management have notably improved due to several coordinated public health initiatives and as per the Pan American Health Organization/WHO directives [12]. Since 2019, the MSPAS has expanded its sentinel surveillance sites and enhanced diagnostic capacity, including the broader availability of enzyme-linked immunosorbent assay and reverse transcription-polymerase chain reaction testing for dengue in regional laboratories (www.mspas.gob.gt). In addition, updated national clinical guidelines for dengue management have been widely disseminated, and health care workers across the country have received training on early detection and case classification based on WHO 2009 criteria. Public health campaigns have also increased community awareness, contributing to earlier care-seeking behavior. These improvements, along with more robust data reporting systems, have collectively strengthened the country’s capacity to monitor and respond to dengue outbreaks more effectively.

The study has limitations that merit comments. First, the surveillance data collected during the COVID-19 epidemic (2020-2022) were insufficient. Second, improved surveillance systems in some regions, such as central Guatemala, may have contributed to higher reported cases, potentially skewing the observed spatial dynamics of dengue incidence. Third, data for children aged under 18 years were available only for specific years (2019, 2023, and 2024), limiting the ability to analyze long-term trends in pediatric dengue cases. However, to the best of our knowledge, this is one of the first reports on temporal and spatial trends of the dengue incidence in Guatemala.

In conclusion, the sharp increase in cases, including those with warning signs, and the doubling of mortality rates in 2024 reflect a growing health crisis, placing immense strain on health care systems and resources. The shift from seasonal outbreaks to year-round transmission complicates prevention and control efforts, requiring sustained interventions. To overcome these challenges, Guatemala can enhance surveillance, adopt year-round vector management, strengthen health care systems, and collaborate globally to manage the growing dengue crisis effectively.

## Declarations of competing interest

The authors have no competing interests to declare.
